# Determination of Minerals in Herbal Infusions Promoting Weight Loss

**DOI:** 10.1007/s12011-016-0790-4

**Published:** 2016-06-29

**Authors:** Wioletta Samolińska, Bożena Kiczorowska, Małgorzata Kwiecień, Elżbieta Rusinek-Prystupa

**Affiliations:** 1 Department of Bromatology and Food Physiology, University of Life Sciences, Akademicka 13, 20-950 Lublin, Poland; 2 Department of Animal Nutrition, University of Life Sciences, Akademicka 13, 20-950 Lublin, Poland; 3 Department of Biochemistry and Toxicology, University of Life Sciences, Akademicka 13, 20-950 Lublin, Poland

**Keywords:** Herbal teas, Weight loss, Macroelements, Microelements, Dietary reference intakes

## Abstract

The study aimed at determination of the mineral composition of slimming herbal teas and estimation of the coverage of their total intake with infusions in women’s daily diet. The content of Na^+^, K^+^, Ca^+2^, Mg^+2^, Zn^+2^, Cu^+2^, Fe^+2^, and Mn^+2^ was determined in infusions and mineralisates obtained from the slimming herbal teas. Among macroelements, the highest content was recorded for Ca—on average 3.73 mg·100 ml^−1^ in its infusion. Mn was a microelement with the highest concentration amounting to 0.20 mg·100 ml^−1^ in the infusion. The investigations revealed that, referring to the dietary reference intakes (DRIs), weight loss herbal infusions cover the recommended daily intake of manganese for women to the highest extent (on average 54 %), which suggests that they can be a major source of this microelement for the organism. Herbal teas only to a slight extent (to approx. 4 %) covered the recommended daily intake of magnesium, sodium, potassium, iron, zinc, copper, and calcium in the daily diet.

## Introduction

Tea is one of the most popular beverages in Poland and in the world. Every year, Poles use on average ca. 1 kg of tea to prepare the beverage, which places Poland among the ten top consumers of tea in the world (eighth place in the world and fourth in Europe) [1]. This beverage owes its popularity to the variety of flavours and the healthy properties of tea associated with the content of many bioactive compounds. The term ‘tea’ is currently used to describe not only black, green, red, or white tea but is also used in reference to herbal infusions obtained from plants other than *Camellia*. ‘Teas’ also denote mixes of dried fruit, herbs, seasonings, and various additions. Frequently, the recipe of such a mix does not contain tea leaves at all. Depending on the composition, they are used, among other applications, in herbal medicine or in weight loss treatments. Such tea infusions are often consumed several times a day [2, 3]. Since consumers are interested in herbal teas enhancing body weight reduction, it is important to determine their nutritive value. This is particularly significant for women, as they choose weight loss therapy more frequently than men do [4].

The study aimed at determination of the mineral composition of slimming herbal teas and evaluation of the coverage of the recommended daily intake of mineral elements by tea infusions in women.

## Material and Methods

### Material

The content of selected macroelements and microelements was determined in the group of weight control products. The studied material comprised nine different kinds of tea from four Polish producers (in 2-g tea bags, 20 in each package) available in Lublin (N 51°14′53″, E 22°34′13″) and purchased in 2012. Table 1 presents data on the composition of the slimming herbal teas (denoted as A, B, C, D, E, F, G, H, and I) and producer’s recommendations of the daily intake. Three packages of each product were analysed. The products were purchased in different shops in order to have material from three different production batches of each herbal tea type. All teas were ahead of their best-before dates. Five tea bags were taken from each purchased package of each kind of tea, and the dried tea leaves were used as a collective sample for further analysis.

**Table 1:** Characteristics of experimental material

Herbal teas	Producer recommendations of infusion intake^*^ ml·day^−1^	Composition
A	600 (3 cups)	Apple, Pu-erh tea, anise, Yerba Mate, mint, lemon, inulin, dog rose, orange, raspberry, aromas
B	400 (2 cups)	Yerba Mate, nettle, dill fruit, apple, pericarp bean, L-carnitine, aroma
C	400 (2 cups)	Hibiscus, apple, Pu-erh tea, Yerba Mate, dog rose, cola nuts, guarana, *Garcinia Cambogia*, L-carnitine
D	600 (3 cups)	Hibiscus, dandelion, dog rose, nettle, horsetail, corn silk, chamomile, chokeberry, ginseng, artichoke, L-carnitine, aroma
E	600 (3 cups)	Pu-erh tea, hibiscus, Yerba Mate, dog rose, L-carnitine, lotus, konjac, aromas
F	600 (3 cups)	Black tea, dog rose, apple, hibiscus, horsetail, Super Citrimax, dandelion, orange, Rooibos, aromas.
G	200 (1 cup)	Senna, hibiscus, mixed fruit (elderberry, apple, black currant, dog rose).
H	600 (3 cups)	Pu-erh tea, hibiscus, elderberry, apple, dog rose, *Garcinia cambogia*
I	200 (1 cup)	Hibiscus, senna

^*^Assuming that one serving of herbal infusion is 200 ml

### Mineral Analysis of Slimming Herbal Tea Samples and their Infusions

In total, 54 samples of nine different types of herbal tea were analysed (dry tea and infusions). Six samples were analysed from each kind of herbal tea. The chemical analysis involved determination of the content of Na^+^, K^+^, Ca^+2^, Mg^+2^, Zn^+2^, Cu^+2^, Fe^+2^, and Mn^+2^ in mineralised tea samples (*n* = 3) and infusions (*n* = 3). The contents of the elements were determined in the tea materials (2 g of herbal material) after incineration in a muffle furnace at 450 °C. The resultant ash was solubilized on crucibles using 6 mol l^−1^ of spectrally pure hydrochloric acid (POCH, Poland). Na and K were analysed using flame atomic emission spectroscopy (FAES) with a flame photometer (Pye Unicam SP 2900, Cambridge, UK) at a wavelength of λ = 589.0 and λ = 766.5 nm, respectively. Ca, Mg, Zn, Cu, Fe, and Mn concentrations were determined using flame atomic absorption spectroscopy (FAAS) with a SOLAAR 939/959 spectrophotometer (Unicam, Cambridge, UK). Calcium was determined at λ = 422.7 nm, magnesium at λ = 285.2 nm, zinc at λ = 213.9 nm, copper at λ = 324.8 nm, iron at λ = 248.3 nm, and manganese at λ = 279.5 nm [5]. In the case of Na and K determinations, cesium chloride (Merck, Poland) was added to the standards and samples as an ionization buffer at a concentration of 0.2 % *w*/*v*. Ca and Mg were analysed by addition of 0.4 % *w*/*v* lanthanum oxide (Merck, Poland), which is a correction buffer that allows binding of the analysed element to the matrix. The percentage of extraction of the elements into the infusion in the course of brewing was determined. The herbal infusion was made by pouring 200 ml of boiling ultrapure water obtained from a HLP 5 system (Hydrolab, Gdansk, Poland) over a 2-g tea sample (loose). Ultrapure water was used in all analyses. The brewing time was approximately 10 min—as recommended by the producer on the package. The infusions were drained, and the residue on the drain was flushed with hot water. The prepared herbal tea infusions (10 ml) were placed in PP tubes and acidified with HNO_3_ (POCH, Poland) to a concentration of 0.25 mol l^−1^ [6]. The concentrations of the elements in the infusions were determined using the methods described above.

The analytical facilities and their quality control system are certified under PN-EN ISO/IEC 17025:2005 [7]. The accuracy of the analytical procedure was verified by an analysis of certified reference materials Mixed Polish Herbs (INCT-MPH-2), manufactured by the Institute of Nuclear Chemistry and Technology (Warsaw, Poland). The recovery levels (*n* = 3) and relative standard deviations (RSD) for the analysed elements were as follows: Na (102.3 %, 6.4 %); K (96.4 %; 7.5 %); Ca (99.7 %, 8.6 %); Mg (100.7 %, 3.9 %); Zn (102.0 %, 4.7 %); Cu (94.6 %, 7.9 %); Fe (94.5 %, 8.2 %); and Mn (96.8 %, 6.9 %).

### Oxalate Analysis in Infusions

Additionally, the content of soluble oxalates was determined in the three infusions for each herbal tea. The manganometric method [8] was adopted from Rusinek et al. [9]. Ten millilitres of the infusions was taken for analysis and transferred to centrifuge tubes. Five millilitres of 5 % CaCl_2_ and 5 ml of acetone were added and mixed. Then, the solutions were cooled at 5 °C for 30 min and centrifuged for 15 min (3000 rpm). The sediment was transferred to the flask with 5 ml 10 % H_2_SO_4_ and heated on a water bath (70 °C) until dissolution. Titration in hot temperature was conducted with a 0.0224 mol l^−1^ solution of KMnO_4_ until pink colour appeared and remained for ca. 1 min.

### Statistical Analysis

The normality and homogeneity of variance data of the mineral composition were tested using the Shapiro-Wilk and Brown-Forsythe tests, respectively. The non-parametric Kruskal-Wallis test (a non-parametric equivalent of one-way analysis of variance) was used to analyse differences in element concentrations in the herbal teas. The performed test showed a normal distribution of the manganese concentrations determined in the herbal tea as well as zinc and iron in the infusions. Detailed comparisons between the groups were conducted using the post hoc Dunn test. All statements of significance were based on a probability of <0.05 and <0.01.

The Pearson correlation coefficient was calculated for the content of oxalate acid and calcium in the infusions. All calculations were performed with statistical software package Statistica version 10 [10].

The intakes of Na, K, Ca, Mg, Zn, Cu, Fe, and Mn with daily tea portions (Table 1) were evaluated using the dietary reference intakes (DRIs) (estimated average requirements—EAR and adequate intakes—AI) for adult women, based on the recommendations of the US National Academy of Sciences (NAS), Institute of Medicine (IOM), and Food and Nutrition Board (FNB) [11] and Polish recommendations of the National Food and Nutrition Institute [12].

## Results and Discussion

### Content of Na, K, Ca, Mg, Zn, Cu, Fe, and Mn in Herbal Teas and their Infusions

As regards macroelements, the herbal teas selected for the study contained the highest amount of calcium, and manganese was a predominant microelement (Table 2). Similar concentrations of elements were determined in the evaluated herbal teas and their infusions. The following contents of minerals were determined for tea as a market product: Ca > Mg > K > Mn > Na > Fe > Zn > Cu (in a descending order), whereas the order in the herbal infusions was Ca > Mg > K > Na > Mn > Zn > Fe > Cu.

**Table 2:** Concentrations of elements in the analysed herbal teas

Herbal teas	Macroelements			Micronutrients		
	mg·100 g^−1^ of market product	mg·100 ml^−1^ of infusion	% of extraction	mg·100 g^−1^ of market product	mg·100 ml^−1^ of infusion	% of extraction
	Sodium (Na)			Zinc (Zn)		
A	49.49^AB^ ± 1.44 (48.05–50.93)	0.42^AB^ ± 0.02 (0.40–0.45)	85.34	23.76^A^ ± 0.69 (23.07–24.45)	0.011^e^ ± 0.001 (0.010–0.011)	4.567
B	27.53^AB^ ± 0.16 (27.37–27.69)	0.26^AB^ ± <0.01 (0.26–0.27)	95.75	11.50^AB^ ± 0.07 (11.43–11.57)	0.027^a^ ± <0.001 (0.027–0.027)	23.57
C	36.10^AB^ ± 1.72 (34.38–37.82)	0.30^AB^ ± 0.03 (0.27–0.33)	82.50	9.18^AB^ ± 0.44 (8.74–9.61)	0.022^bc^ ± 0.002 (0.020–0.024)	24.04
D	44.20^AB^ ± 3.74 (40.46–47.94)	0.40^AB^ ± 0.07 (0.33–0.47)	90.04	9.38^AB^ ± 0.79 (8.59–10.17)	0.020^c^ ± 0.003 (0.016–0.023)	20.81
E	24.87^B^ ± 0.13 (24.74–25.01)	0.24^B^ ± <0.01 (0.23–0.24)	94.73	5.03^B^ ± 0.33 (5.00–5.05)	0.019^c^ ± <0.001 (0.019–0.020)	38.73
F	39.27^AB^ ± 0.55 (38.72–39.82)	0.35^AB^ ± 0.01 (0.34–0.35)	87.89	2.93^B^ ± 0.04 (2.89–2.97)	0.013^e^ ± <0.001 (0.012–0.013)	43.57
G	38.89^AB^ ± 1.28 (37.61–40.17)	0.33^AB^ ± 0.02 (0.30–0.35)	83.79	3.10^AB^ ± 0.10 (3.00–3.20)	0.016^de^ ± 0.001 (0.015–0.017)	50.37
H	88.45^A^ ± 1.20 (87.25–89.65)	0.72^A^ ± 0.02 (0.70–0.74)	80.95	6.31^AB^ ± 0.09 (6.22–6.39)	0.018^d^ ± <0.001 (0.017–0.018)	28.37
I	49.26^AB^ ± 1.19 (48.07–50.44)	0.43^AB^ ± 0.02 (0.41–0.45)	87.66	3.16^AB^ ± 0.08 (3.08–3.23)	0.023^b^ ± 0.001 (0.022–0.024)	73.71
Mean ± SD	44.23 ± 17.97	0.38 ± 0.14	87.63 ± 5.65	8.26 ± 6.35	0.019 ± 0.005	34.19 ± 19.35
	Potassium (K)			Copper (Cu)		
A	102.39^B^ ± 2.97 (99.41–105.36)	0.95^AB^ ± 0.06 (0.90–1.01)	93.09	1.17^AB^ ± 0.03 (1.14–1.21)	0.001^B^ ± <0.001 (0.001–0.001)	11.04
B	124.84^AB^ ± 0.73 (124.11–125.58)	1.12^AB^ ± 0.01 (1.10–1.13)	89.41	1.00^AB^ ± 0.01 (1.00–1.01)	0.007^A^ ± 0.001 (0.007–0.007)	70.11
C	211.11^A^ ± 10.05 (201.06–221.17)	1.78^A^ ± 0.17 (1.61–1.95)	84.21	1.07^AB^ ± 0.05 (1.02–1.13)	0.003^AB^ ± <0.001 (0.003–0.003)	28.42
D	171.64^AB^ ± 14.51 (157.12–186.15)	1.50^AB^ ± 0.25 (1.25–1.75)	86.92	0.83^AB^ ± 0.07 (0.76–0.90)	0.003^AB^ ± <0.001 (0.003–0.004)	39.43
E	138.62^AB^ ± 0.75 (137.87–139.37)	1.15^AB^ ± 0.01 (1.14–1.16)	82.82	1.85^A^ ± 0.01 (1.84–1.86)	0.002^AB^ ± <0.001 (0.002–0.003)	13.41
F	134.25^AB^ ± 1.88 (132.36–136.13)	1.14^AB^ ± 0.03 (1.10–1.17)	84.57	0.95^AB^ ± 0.01 (0.94–0.96)	0.004^AB^ ± <0.001 (0.004–0.004)	41.19
G	100.14^B^ ± 3.30 (96.84–103.44)	0.86^B^ ± 0.06 (0.81–0.92)	86.04	0.42^B^ ± 0.01 (0.40–0.43)	0.002^AB^ ± <0.001 (0.002–0.002)	42.02
H	127.77^AB^ ± 1.73 (126.04–129.50)	1.14^AB^ ± 0.03 (1.11–1.17)	89.42	1.05^AB^ ± 0.01 (1.04–1.06)	0.002^AB^ ± <0.001 (0.002–0.002)	21.29
I	129.33^AB^ ± 3.12 (126.21–132.45)	1.11^AB^ ± 0.05 (1.06–1.17)	86.11	0.43^AB^ ± 0.01 (0.42–0.44)	0.002^AB^ ± <0.001 (0.002–0.002)	41.07
Mean ± SD	137.79 ± 33.59	1.19 ± 0.28	86.95 ± 4.07	0.97 ± 0.41	0.003 ± 0.002	34.22 ± 17.50
	Calcium (Ca)			Iron (Fe)		
A	381.64^AB^ ± 11.08 (370.57–392.72)	0.76^F^ ± 0.04 (0.72–0.81)	19.98	20.78^AB^ ± 0.60 (20.18–21.39)	0.006^D^ ± <0.001 (0.006–0.007)	3.10
B	805.68^AB^ ± 4.74 (800.94–810.42)	1.71^E^ ± 0.02 (1.69–1.73)	21.21	13.41^B^ ± 0.08 (13.33–13.48)	0.001^F^ ± <0.001 (0.001–0.002)	1.12
C	849.78^AB^ ± 40.47 (809.31–890.24)	4.67^C^ ± 0.44 (4.23–5.12)	54.88	22.00^AB^ ± 1.051 (20.95–23.04)	0.011^C^ ± 0.001 (0.010–0.012)	5.09
D	799.89^AB^ ± 67.64 (732.25–867.54)	4.35^C^ ± 0.73 (3.63–5.10)	54.17	34.64^AB^ ± 2.93 (31.71–37.56)	0.016^B^ ± 0.003 (0.013–0.019)	4.61
E	266.90^B^ ± 1.45 (265.45–268.34)	1.30^EF^ ± 0.01 (1.28–1.31)	48.64	37.48^A^ ± 0.20 (37.28–37.68)	0.014^BC^ ± <0.001 (0.014–0.014)	3.65
F	759.71^AB^ ± 10.67 (749.04–770.37)	3.16^D^ ± 0.09 (3.07–3.25)	41.62	17.64^AB^ ± 0.25 (17.40–17.89)	0.010^C^ ± <0.001 (0.010–0.010)	5.54
G	864.86^AB^ ± 28.48 (836.38–893.34)	4.48^C^ ± 0.29 (4.18–4.77)	51.72	17.60^AB^ ± 0.58 (17.02–18.18)	0.012^C^ ± 0.001 (0.012–0.013)	7.02
H	1236.03^AB^ ± 16.74 (1219.29–1252.76)	5.82^B^ ± 0.16 (5.67–5.98)	47.12	20.57^AB^ ± 0.28 (20.29–20.85)	0.016^B^ ± <0.001 (0.016–0.017)	7.95
I	1407.07^A^ ± 33.94 (1373.13–1441.02)	7.33^A^ ± 0.35 (6.97–7.68)	52.04	13.78^AB^ ± 0.33 (13.45–14.12)	0.021^A^ ± 0.001 (0.020–0.022)	15.46
Mean ± SD	819.06 ± 344.53	3.73 ± 2.12	43.49 ± 13.15	21.99 ± 8.25	0.012 ± 0.006	5.95 ± 3.95
	Magnesium (Mg)			Manganese (Mn)		
A	233.52^AB^ ± 6.78 (226.74–240.30)	1.12^B^ ± 0.07 (1.06–1.19)	48.11	38.90^FG^ ± 1.13 (37.77–40.03)	0.115^AB^ ± 0.007 (0.108–0.121)	29.48
B	474.45^A^ ± 2.79 (471.65–477.24)	2.89^A^ ± 0.03 (2.86–2.93)	60.93	69.28^B^ ± 0.41 (68.87–69.69)	0.324^AB^ ± 0.004 (0.320–0.327)	46.69
C	248.37^AB^ ± 11.83 (236.55–260.20)	2.04^AB^ ± 0.19 (1.85–2.23)	81.98	55.42^DE^ ± 2.64 (52.78–58.06)	0.332^A^ ± 0.032 (0.301–0.364)	59.88
D	261.19^AB^ ± 22.09 (239.10–283.28)	1.92^AB^ ± 0.32 (1.60–2.24)	73.05	32.68^G^ ± 2.76 (29.92–35.44)	0.104^AB^ ± 0.017 (0.087–0.122)	31.65
E	250.89^AB^ ± 1.36 (249.53–252.25)	1.27^AB^ ± 0.01 (1.25–1.28)	50.51	73.05^A^ ± 0.40 (72.66–73.45)	0.299^AB^ ± 0.003 (0.296–0.302)	40.93
F	173.75^B^ ± 2.44 (171.31–176.19)	1.36^AB^ ± 0.04 (1.32–1.40)	78.19	50.70^E^ ± 0.71 (49.99–51.42)	0.145^AB^ ± 0.004 (0.141–0.149)	28.59
G	224.97^AB^ ± 7.41 (217.56–232.38)	1.74^AB^ ± 0.11 (1.63–1.86)	77.49	14.82^I^ ± 0.49 (14.33–15.31)	0.089^B^ ± 0.006 (0.083–0.095)	60.15
H	210.51^AB^ ± 2.85 (207.66–213.36)	1.61^AB^ ± 0.04 (1.57–1.65)	76.47	59.23^C^ ± 0.80 (58.43–60.03)	0.291^AB^ ± 0.008 (0.283–0.298)	49.05
I	312.68^AB^ ± 7.54 (305.13–320.22)	2.77^AB^ ± 0.13 (2.63–2.90)	88.41	28.34^H^ ± 0.68 (27.66–29.03)	0.142^AB^ ± 0.007 (0.135–0.149)	50.14
Mean ± SD	265.59 ± 83.93	1.86 ± 0.61	70.57 ± 13.76	46.94 ± 18.84	0.204 ± 0.100	44.06 ± 11.81

Arithmetic mean (*n* = 3) ± standard deviations (SD) and range are shown

a, b, c, d, e represent statistical differences *P* < 0.05; A, B, C, D, E, F, G, H, I represent statistical differences *P* < 0.01

The results of our investigations were compared with results of similar studies involving the analysis of the elemental composition of teas (black, green, red), herbal teas, and slimming herbal teas.

The determined content of calcium was from 266.90 to 1407.07 mg·100 g^−1^ and that of magnesium ranged from 173.75 to 474.45 mg·100 g^−1^ of the market product (*P* < 0.01) (Table 2). The results obtained in this study and by other authors suggest that Ca and Mg are present in medicines of plant origin and teas with varied composition (herbs, teas, fruits, bioactive ingredients) in considerable amounts [2, 13, 14]. *Garcinia cambogia* widely used as a component of slimming formulations can be an example*.* Commercial samples of *G. cambogia* extracts contain calcium salt of HCA ((−)-hydroxycitric acid) for its stability [15]. In this study, its content in herbal teas C and H may contribute to the increased calcium amounts. The highest content of this element was noted in herbal tea I (*P* < 0.01), which was probably attributed to the presence of senna (*Senna alexandrina* Mill.), which contains 898.9-mg calcium in 100 g^−1^, as indicated by Ebrahim et al. [16].

In the presented study, the amount of potassium in the herbal teas did not exceed 222 mg·100 g^−1^ and that of sodium was lower than 90 mg per 100 g of the market product. Available literature indicates that the level of potassium and sodium in teas varies considerably depending on the origin of the plant material. It can even vary for the same type of tea [13, 17, 18]. The highest potassium content (*P* < 0.01) was determined in herbal tea C, which can be associated with the presence of kola nuts (*Cola* spp.) in its composition, which contain substantial amounts of this element (348.47 mg·100 g^−1^ d.m.) [19].

The content of zinc was determined in the weight loss herbal teas in a broad range from 2.93 to 23.76 mg·100 g^−1^, and the content of iron was from 13.41 to 37.48 mg·100 g^−1^ (*P* < 0.01). Such a span can be connected with the rich ingredient composition of the studied products. Other authors reported less diverse results concerning the content of these elements in slimming teas [14, 20]. In the infusion of herbal tea B, with Yerba mate (*Ilex paraguariensis* A. St.-Hil.) as the main ingredient, the highest zinc content was noted (*P* < 0.05). This was confirmed by the investigations conducted by Bragança [21], who reported slightly higher zinc contents in infusions of only this type of tea (0.041 to 0.10 mg·100 ml^−1^).

In turn, the amount of copper recorded in our study (0.42 to 1.85 mg·100 g^−1^) was similar to that reported by other authors (from 0.72 to 1.54 mg·100 g^−1^) [14, 20].

The content of manganese was determined in a range from 14.82 to 73.05 mg·100 g^−1^ of herbal tea (*P* < 0.01). The results were close to those reported by other authors [14, 20, 22]. Similarly, in the case of manganese, the presence of Yerba mate in the composition may have contributed to the higher amounts of this element in the infusions of herbal teas C, B, and E (0.33–0.30 mg·100 ml^−1^). Considerable amounts of Mn in Yerba mate infusions (0.27–0.70 mg·100 ml^−1^) were also reported by Bragança [21].

The degree of extraction of the elements into the infusion was as follows: Na > K > Mg > Mn > Ca > Cu > Zn > Fe (in a descending order) (Table 2). The highest percentage of extraction into the infusion was determined for sodium and potassium (over 80 %), and nearly half of the manganese was transferred to the infusion. On average, up to 40 % of copper and zinc were extracted into the beverage. The weakest transfer into the herbal tea was noted for iron (on average less than 6 %). Similar results were reported by other authors [2, 14, 20]. For manganese and calcium, the average degree of extraction was higher than that recorded by the abovementioned authors. For manganese, it was above 70 % (48.11–88.41 %) and for calcium above 40 % (19.98–54.88 %). A similar level of solubility of the analysed elements in slimming herbal teas was observed by Szymczycha-Madeja et al. [14], who also reported a high percentage of transfer of manganese into the infusion (72.9–88.4 %) and a moderate percentage for calcium (21.5–54.5 %).

The degree of extraction of minerals into the infusion is modified by many factors and is determined, among other things, by the method of infusion preparation, brewing time, type of plant material used, tea components, and the form of market product (tea leaves or tea bags). The content of such compounds as tannins or oxalic acid in tea can reduce the absorption of minerals as well [9, 23]. In the presented study, the following content of oxalic acid was determined in the respective teas—17.11 mg (tea A), 34.21 mg (B), 24.02 mg (C), 24.06 mg (D), 60.01 mg (E), 26.01 mg (F), 22.02 mg (G), 16.01 mg (H), and 18.05 mg (I) per 100 ml of tea. These values were negatively correlated with the concentration of calcium (*r* = −0.539). A higher content of oxalic acid was noted by Rusinek [9], who determined the content of this anti-nutritive component in black tea (on average 115.68 mg·100 ml^−1^), green tea (87.64 mg·100 ml^−1^), and red tea (101.91 mg·100 ml^−1^). The author also noted a lower content of oxalates in infusions from bagged tea compared to loose-leaf tea and observed a similar relationship for teas with an admixture of other plant components (apple, hibiscus, briar, or grapefruit), compared with teas from *Camellia* genus plants. Herbal tea E characterised by the highest content of oxalates contained Pu-erh tea as well as konjac (*Amorphophallus muelleri* Blume), which probably contributed to the high level of these compounds in the infusion, since the plant contains substantial amounts thereof in all its parts [24]. Besides plants from the genus *Camellia*, the stinging nettle (*Urtica dioica* L.) may have been a rich source of oxalic acid in the analysed beverages (herbal tea B). This is confirmed by investigations conducted by other authors. Sperkowska and Bazylak [25] determined a level of 59.91 mg of soluble oxalates per 100 ml of nettle infusion. Herbal tea B contained fennel seeds, which may have increased the content of water-soluble oxalates in the infusion. As reported by Al-Wahsh [26], seeds of fennel (*Foeniculum vulgare* Mill.) contain 1086 mg of total oxalate in 100 g^−1^ dry weight and 194 mg of soluble oxalates in 100 g^−1^ dry weight. Another ingredient of the analysed herbal teas, *G. cambogia*, is characterised by a twofold higher content of water-soluble oxalates (137.52 mg·100 g^−1^ d.m) [27]. However, the proportion of *G. cambogia* in the analysed beverages is low; hence, its effect on the content of soluble oxalates in teas C and H is probably insignificant.

Small amounts of soluble oxalates are also contained in the fruit components of the analysed types of herbal tea. Their content in apples is 2.2 mg·100 g^−1^ fresh weight and in oranges 2.9 mg·100 g^−1^ fresh weight [26]. The literature does not provide data on the content of soluble oxalates in the other plant ingredients of the analysed herbal tea, i.e. in hibiscus, rosehip fruit, lotus flowers, and others, which makes the interpretation of the results difficult.

Teas, herbal, and/or fruit teas also contain non-essential and toxic metals, e.g. Al, Cd, Ni, or Pb, which are extracted into the infusion and may pose a health hazard to consumers. Commission Regulation (EC) no. 629/2008 of 2 July 2008, amending Regulation (EC) no. 1881/2006 set maximum levels for certain metals in food supplements: lead (3.0 mg·kg^−1^), cadmium (1.0 mg·kg^−1^), and mercury (0.10 mg·kg^−1^) [28]. The maximum allowable level of these elements applies to dietary supplements in the commercial form. In the investigations carried out by Jeszka-Skowron [29], the Al content in green tea infusion and black tea infusion was negligible, i.e. 0.008 and 0.011 %, respectively. In the study reported by Łozak [20], in which the mineral composition of slimming herbal tea was analysed, the concentrations of cadmium and lead in all samples were below the EC maximum level for Cd and Pb. In this study, the levels of these heavy metals were not determined.

### Dietary Intake of Minerals from Slimming Herbal Tea Infusions

The daily intake of the studied elements from the infusions of weight loss herbal teas was analysed and presented as a percentage of dietary reference intakes (DRIs) for adult women [11, 12] (Table 3). In this study, the bioavailability of elements from the infusions in vivo was not determined. We only analysed the effect of soluble oxalates on calcium availability from the infusions. Therefore, total concentrations of other elements in the herbal infusions were compared with the DRI values. The elements can be divided into three groups, depending on the degree of coverage of the organism’s demand: elements present in tea in inconsiderable amounts, such as iron, sodium, and potassium, covered the recommended daily intake only up to ca. 1 % (0.04–1.23 %). The second group comprises components such as magnesium, copper, calcium, and zinc, the requirement for which was covered by the tea infusions at a low level—up to ca. 4 % (0.46–4.37 %). The third group included only manganese present in the infusions in considerable amounts and covering the standard nutrition requirement in a wide range from 9.91 to 99.68 %. On average, it covered more than a half of the dietary reference intake (54.36 %) for adult women. In their study, Powell et al. [30] analysed the bioavailability of manganese from tea infusions by incubation thereof in human gastric acid (37 °C, 1 h). A single serving of tea (225 ml) covered 10 % of the daily intake of this element in a potentially bioavailable form. Reference literature reports that manganese is the only element occurring in tea—mostly black tea—in considerable amounts from the point of view of nutrition [14, 20, 30]. In the daily diet, manganese is mainly consumed with food products of plant origin and with water, but only a small percentage (1–4 %) is absorbed in the alimentary tract [31]. This element also takes part in the formation of bone and connective tissue and is a component or an activator of enzymes. It also participates in the metabolism of amino acids, carbohydrates, and cholesterol [32]. Some epidemiological studies report adverse neurological effects of exposure to very high levels of manganese in drinking water [30].

**Table 3:** The calculated daily intake of elements with herbal infusions and realization of DRI values

Herbal teas	Daily intake/ DRI realization	Element							
		Na	K	Ca	Mg	Zn	Cu	Fe	Mn
DRI^*^	mg/day	1500	4700	800	265	6.80	0.70	8.00	1.80
A	mg/day	2.54	5.72	4.58	6.74	0.07	0.01	0.04	0.69
	%	0.17	0.12	0.57	2.54	0.96	1.11	0.48	38.25
B	mg/day	1.05	4.46	6.84	11.56	0.11	0.03	0.01	1.29
	%	0.07	0.09	0.85	4.36	1.59	4.02	0.07	71.89
C	mg/day	1.19	7.12	18.68	8.16	0.09	0.01	0.04	1.33
	%	0.08	0.15	2.34	3.08	1.30	1.75	0.56	73.86
D	mg/day	2.40	8.99	26.12	11.50	0.12	0.02	0.10	0.62
	%	0.16	0.19	3.27	4.34	1.73	2.82	1.20	34.64
E	mg/day	1.41	6.89	7.79	7.60	0.12	0.01	0.08	1.41
	%	0.09	0.15	0.97	2.87	1.72	2.12	1.03	99.68
F	mg/day	2.07	6.81	18.97	8.15	0.08	0.02	0.06	0.87
	%	0.14	0.14	2.37	3.08	1.13	3.35	0.73	48.32
G	mg/day	0.65	1.72	8.95	3.49	0.03	0.004	0.02	0.18
	%	0.04	0.04	1.12	1.32	0.46	0.50	0.31	9.91
H	mg/day	4.30	6.86	34.95	9.66	0.11	0.01	0.10	1.74
	%	0.29	0.15	4.37	3.65	1.58	1.92	1.23	96.85
I	mg/day	0.86	2.23	14.65	5.53	0.05	0.004	0.04	0.28
	%	0.06	0.05	1.83	2.09	0.68	0.50	0.53	15.80
Mean	mg/day	1.83	5.65	15.73	8.04	0.08	0.01	0.05	0.98
	%	0.12	0.12	1.97	3.04	1.24	2.01	0.68	54.36

^*^DRI [11, 12]

Women tend to absorb more manganese than men do [31, 32]. Given this fact and the considerable content of this element in herbal infusions, the percentage share of manganese was also calculated with reference to the standard upper level (UL), i.e. 11 mg day^−1^ for an adult woman [32]. This standard is the maximum biologically tolerable customary level of intake of the mineral component that has no adverse effect on health in 97.5 % of people in a population [11, 12]. The corresponding values in the presented study ranged from 1.622 % (herbal infusion G) to 16.31 % (herbal infusion E), reaching on average 8.90 % of the standard UL, which indicates that slimming herbal teas can be safely used on a daily basis. To fully assess a given food product or beverage as a source of elements in the diet, the total contents of elements not only in vitro but also in vivo should be determined. This would facilitate absorption of these elements in the digestive tract, which is dependent on their bioavailability. However, this type of research is conducted extremely rarely, as it is cost- and time-inefficient and sometimes disputable [30, 33, 34].

## Conclusion

The studied slimming herbal teas contained different amounts of minerals. Calcium and magnesium were the predominant elements determined therein and manganese and iron were the most abundant microelements. Weight loss herbal infusions cover the recommended daily intake at a low level—up to ca. 4 %. In turn, the high content of manganese and the coverage of, on average, more than half of the dietary reference intake of this element, suggest that slimming herbal infusions can be a major source of this element in the daily diet.
